# Beneficial Effect of Oligofructose-Enriched Inulin on Vitamin D and E Status in Children with Celiac Disease on a Long-Term Gluten-Free Diet: A Preliminary Randomized, Placebo-Controlled Nutritional Intervention Study

**DOI:** 10.3390/nu10111768

**Published:** 2018-11-15

**Authors:** Natalia Drabińska, Urszula Krupa-Kozak, Paweł Abramowicz, Elżbieta Jarocka-Cyrta

**Affiliations:** 1 Department of Chemistry and Biodynamics of Food, Institute of Animal Reproduction and Food Research of Polish Academy of Sciences, Tuwima 10 Str., 10-748 Olsztyn, Poland; n.drabinska@pan.olsztyn.pl; 2 Department of Pediatrics, Rheumatology, Immunology and Metabolic Bone Diseases, Medical University of Białystok, Waszyngtona 17 Str., 15-274 Białystok, Poland; pabram@o2.pl; 3 Department of Pediatrics, Gastroenterology and Nutrition, Collegium Medicum, University of Warmia & Mazury, Oczapowskiego 2 Str., 10-719 Olsztyn, Poland; ejarocka@op.pl

**Keywords:** celiac disease, inulin, prebiotics, gluten-free diet, dietary intervention, vitamin D, vitamin E

## Abstract

Prebiotics have been shown to improve absorption of some nutrients, including vitamins. This pilot study evaluated the effect of the prebiotic oligofructose-enriched inulin (Synergy 1) on fat-soluble vitamins status, parathormone, and calcium-related elements in pediatric celiac disease (CD) patients (*n* = 34) on a strict gluten-free diet (GFD). Participants were randomized into a group receiving 10 g of Synergy 1 or placebo (maltodextrin) together with a GFD. At baseline and after 3 months of intervention, 25-hydroxyvitamin D [25(OH)D], parathormone, vitamin E and A, calcium, phosphate, magnesium, total protein, and albumin were determined. Concentration of 25(OH)D increased significantly (*p* < 0.05) by 42% in CD patients receiving Synergy 1 in GFD, whereas no change was observed in placebo. Vitamin D status reached an optimal level in 46% of patients receiving Synergy 1. No significant difference in parathormone, calcium, and phosphate levels was observed. Concentration of vitamin E increased significantly (*p* < 0.05) by 19% in patients receiving Synergy 1, but not in the placebo. Vitamin A levels were not changed. Supplementation of GFD with Synergy 1 improved vitamin D and vitamin E status in children and adolescents with CD and could be considered a novel complementary method of management of fat-soluble vitamins deficiency in pediatric CD patients.

## 1. Introduction

Over the previous decades, the role of vitamins in the prevention or treatment of diseases has been acknowledged [1]. The general classification of vitamins is based on their solubility in water or fats. Fat-soluble vitamins include vitamins A, D, E, and K. Vitamins A and E have antioxidant and anti-inflammatory activity, while vitamins D and K are among others involved in bone metabolism [2]. Vitamin D deficiency can lead to serious consequences, including impairment of bone mineralization; metabolic syndrome; autoimmune disorders; and psychiatric, neurological, and cardiovascular diseases. Besides this, vitamin D status is inversely correlated with the incidence of many diseases including cancers, cardiovascular diseases, and neurodegenerative diseases [3,4].

Celiac disease (CD) is a chronic, immune-mediated small-intestinal enteropathy observed in genetically predisposed individuals, initiated by exposure to gluten [5]. Since CD is characterized by gluten-induced villous atrophy, often accompanied by malabsorption, vitamin D deficiency is commonly observed in this condition, even in patients adherent to a gluten-free diet (GFD). Eighty percent of vitamin D is synthesized endogenously in skin upon exposure to ultraviolet radiation, while 20% is derived from dietary sources and absorbed with fat in the small intestine [6]. A reduction of the small-intestinal absorptive mucosal surface, which can be secondary to various diseases including CD, is one of the possible factors leading to hypovitaminosis D. In addition, steatorrhea, which occurs in untreated CD, may impair small-intestinal reabsorption of 25-hydroxyvitamin D [25(OH)D] into enterohepatic circulation. Vitamin D deficiency and resulting hypocalcaemia can lead to secondary hyperparathyroidism, which in turn stimulates bone turnover, with potential clinical consequences such as reduced bone mineral density, osteoporosis, or rickets [7,8,9]. In addition to vitamin D deficiency, other vitamin levels, including those of vitamins A and E, have been found to be decreased in CD patients [10,11].

One possible strategy to avoid nutrient deficiencies in CD patients is the appropriate dietary supplementation. According to the most recent definition proposed by the International Scientific Association of Probiotics and Prebiotics in 2017, prebiotics are substrates that are selectively utilized by host microorganisms conferring a health benefit [12]. Prebiotics can have beneficial effects on vitamin and mineral absorption. In particular, inulin-type fructans have been shown to alleviate calcium deficiencies in young adolescents [13]. Prebiotic Synergy 1, a mixture of short-chain fructooligosaccharides and long-chain inulin in a 1:1 ratio, has been proven to be effective in improving calcium and magnesium deficiencies in postmenopausal women [8]. However, to our knowledge, the effects of supplementation of a GFD in pediatric CD patients with prebiotics have not been studied yet. Therefore, the objective of this study was to evaluate the effect of oligofructose-enriched inulin (Synergy 1) on fat-soluble vitamins, parathormone, and minerals status in children with CD on the GFD.

## 2. Materials and Methods

### 2.1. Study Protocol

A pilot, randomized, placebo-controlled nutritional intervention was conducted in 34 children aged 4–17 years with CD on a strict GFD. The children were patients of the Department of Pediatrics, Gastroenterology and Nutrition of Children’s Hospital in Olsztyn. The clinical trial was registered on http://www.clinicaltrials.gov (No: NCT03064997) and conducted between January and June 2016. The full study protocol was described in our previous study [14]. All participants met the inclusion criteria: CD confirmed by serological, genetic, and biopsy analyses; and GFD for at least 6 months. The exclusion criteria included use of antibiotics in the month preceding the study; use of medication for osteoporosis (bisphosphonates, calcium calcitonin); use of probiotics, prebiotics, or dietary fiber supplements; poor or average overall health; current enrolment in another clinical trial; and recent surgery. Participants were randomly assigned into two groups: a group receiving 10 g of prebiotic oligofructose-enriched inulin (Synergy 1; Orafti^®^, Beneo, Belgium) daily and a placebo group receiving maltodextrin. Stratified randomization based on gender and age was conducted to produce comparable groups. The intervention lasted 3 months in each patient. Study products (prebiotic or placebo) were provided to participants during the enrolment visit. Participants were instructed to record a daily intake of the provided study product and any side effects during the trial on the observation chart provided to each participant at the first visit. Before the intervention, all subjects were receiving vitamin D supplements in a dose consistent with Polish guidelines (800 or 1000 IU per day) [15] and, importantly, this remained unchanged during the trial. Blood and urine samples for biochemical measurements were collected at baseline and on the last day of the study. Patients and their families, clinicians, and laboratory staff were all blinded to the results of the randomization and to baseline laboratory results.

### 2.2. Ethical Statement

Parents and caregivers of participants were informed about the potential benefits and risks of the dietary intervention and signed the written informed consent on the first check-up visit. This study was approved by the Bioethics Committee of the Faculty of Medicine of the University of Warmia and Mazury in Olsztyn, Poland (decision No. 23/2015 of 16 June 2015). The study protocol and all procedures performed in this study involving human participants were done in accordance with the ethical principles of the World Medical Association (WMA) 1964 Declaration of Helsinki and its later amendments or comparable ethical standards.

### 2.3. Assays for Calciotropic Hormones

The plasma concentration of 25(OH)D was determined using an ELISA kit (DIAsource ImmunoAssays S.A., Louvain-la-Neuve, Belgium). The limit of detection (LOD) and the limit of quantification (LOQ) for this assay were 2.81 and 4.39 ng/mL, respectively. The intra-assay precision provided by the manufacturer was <7.8%.

The plasma concentration of parathormone was determined using a human Intact Parathyroid Hormone EASIA Kit (DIAsource ImmunaAssay S.A., Louvain-la-Neuve, Belgium). The LOD of this kit was 2 pg/mL. The intra-assay precision provided by the manufacturer was <2.0%. Optical density measurements for both assays were determined using a Biochrom Asys UVM340 Microplate Reader (Biochrom Ltd., Cambridge, UK).

### 2.4. Biochemical Measurements in Blood and Urine

Calcium (Ca), phosphate (P), magnesium (Mg), albumin, and total protein concentrations were measured in serum. Urinary excretion of Ca, P, and Mg was determined in the first morning urine sample and expressed as mg/mg of creatinine (mg/mg Cr). All measurements were performed according to a standard automated method using a Cobas analyzer equipped with Cobas MIRA Plus (Roche Diagnostics, Warsaw, Poland).

### 2.5. Measurement of Vitamins A and E

The plasma concentrations of vitamins A and E were analyzed using a Vitamins A and E in Serum/Plasma—HPLC kit (Chromsystems Instruments & Chemicals GmbH, Gräfelfing, Germany). Briefly, 200 μL of plasma was mixed with 20 μL of internal standard and 25 μL of precipitation reagent I. After vortexing, 400 μL of precipitation reagent II was added and mixed for 30 s. The mixture was centrifuged, and 50 μL of supernatant was injected into the high-performance liquid chromatography (HPLC) system. Separation was performed in an HPLC system with an autosampler (LC-20) and an SPD-M20A DAD detector (Shimadzu, Kyoto, Japan). The compounds were separated in the isocratic mode using a Chromsystems HPLC column provided by the manufacturer with a flow rate of 1.5 mL/min of mobile phase (Chromsystems Instruments & Chemicals GmbH, Gräfelfing, Germany). The concentrations of vitamins were calculated using external standards for individual compounds and normalized in response to internal standard. The method was linear from LOQ estimated as 0.02 and 0.50 mg/L up to 2.25 and 45.00 mg/L for vitamin A and vitamin E, respectively.

### 2.6. Statistical Analysis

All of the analyses described below were carried out using Statistica 12 software (StatSoft, Tulsa, OK, USA). Results were considered statistically significant at the 5% critical level (*p* < 0.05). Normality of quantitative variables was tested by the Shapiro–Wilk test. Quantitative variables with a normal distribution were expressed as mean ± SD, while quantitative variables which showed a non-normal distribution were expressed as median (P25–P75). Differences in characteristics between groups were tested with the parametric Student’s *t*-test or the nonparametric Mann–Whitney U test. Differences within groups before and after intervention were determined with Student’s *t*-test for paired samples or the Wilcoxon test, as appropriate.

## 3. Results

At baseline, the demographic and anthropometric characteristics as well as all analyzed parameters did not differ between both experimental groups (Table 1 and Table 2). From a group of 34 children—Synergy 1 group (*n* = 18; average age: 10 years (range: 5–17); 11 girls) and placebo group (*n* = 16; average age: 10 years (range: 4–16); 10 girls)—30 children were retained for final analysis. Four children were excluded: two because of antibiotic intake during intervention and two because of compliance to study product intake lower than 80% or a different reason.

**Figure 1:**
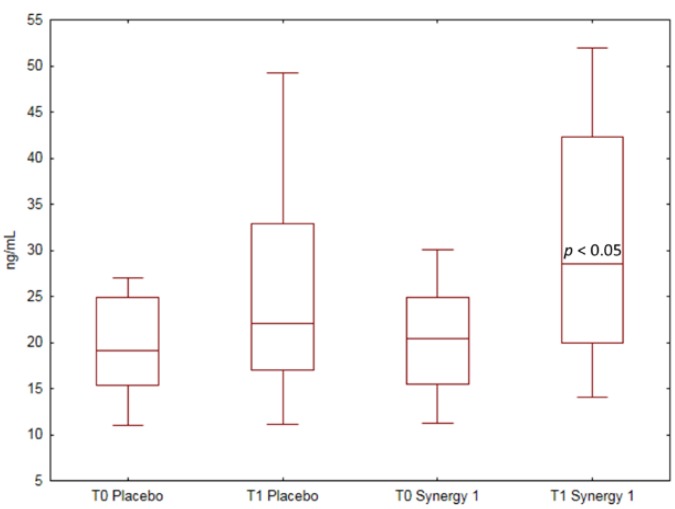
Box-and-whisker plots of 25(OH)D plasma concentration comparison between groups supplemented with Synergy 1 and placebo at baseline (T0) and after 3 months of supplementation (T1) using the Mann–Whitney test. Data are expressed as median (horizontal line), percentiles (box), and range (whisker).

The values of 25(OH)D concentration in both experimental groups before and after the intervention are presented in Figure 1. At baseline, the median 25(OH)D plasma concentration was 20.36 ng/mL (15.48–24.86) in the Synergy 1 group and 19.10 ng/mL (15.30–24.87) in the placebo group, and values did not differ statistically (*p* = 0.603). Before the intervention, a suboptimal level of vitamin D (20–30 ng/mL) was determined in 39% and 33% of CD patients from the Synergy 1 and placebo group, respectively, while deficiency (<20 ng/mL) was noted in 44% and 60% of CD patients from the Synergy 1 and placebo group, respectively.

After 3 months of dietary intervention with Synergy 1, a significant increase (*p* = 0.012) by 42% in the median plasma concentration of vitamin D was observed, reaching 28.57 ng/mL (19.94–42.27). Conversely, in the placebo group, no significant change was observed, and the median vitamin D plasma concentration after intervention was 22.11 ng/mL (17.02–32.90). The percentage of CD patients with optimal 25(OH)D status in the Synergy 1 group increased from 17 to 46% after the intervention.

The results of serum and urine calcium, magnesium, phosphate, total protein, albumin, and parathormone concentration measurements are presented in Table 2. Parathormone concentration did not differ between both groups at baseline and did not change following the intervention. Supplementation of a GFD with Synergy 1 did not affect serum Ca, Mg, or P levels (Table 2). Urinary excretion of minerals was also not affected by the intervention.

At baseline, five children in the Synergy 1 group and one child in the placebo group were found to have serum calcium levels below the lower normal limit (<9.0 mg/dL). After intervention, among these children with hypocalcaemia at baseline, calcium levels were within the normal range in four children from the Synergy 1 group and in the one child from the placebo group.

**Figure 2:**
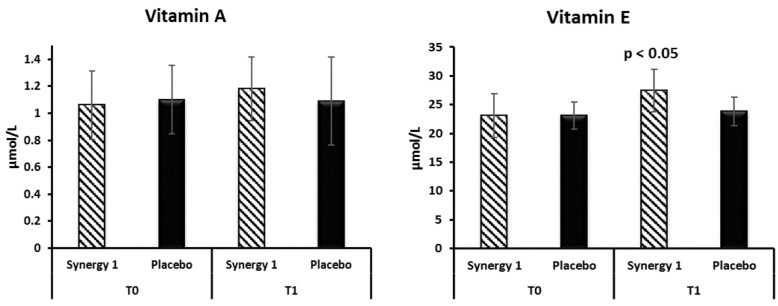
Plasma concentrations of vitamin A and E in individual participants before (T0) and after the intervention (T1), expressed as mean ± SD.

The plasma concentrations of vitamins E and A in both experimental groups before and after the intervention are presented in Figure 2. No difference in vitamin E levels between groups was observed at baseline, with values of 23.09 ± 3.79 and 23.11 ± 2.31 µmol/L in the Synergy 1 and placebo group, respectively. After the intervention, a significant increase (*p* = 0.031) by 19% in vitamin E plasma concentration was observed in the Synergy 1 group, reaching a mean of 27.46 ± 3.73 µmol/L. In the placebo group, vitamin E levels after intervention remained similar to baseline (23.81 ± 2.50 µmol/L). No changes in vitamin A plasma concentration were observed before and after the intervention in both experimental groups; nevertheless, an increasing tendency was observed in the Synergy 1 group (Figure 2).

## 4. Discussion

The main mode of action of prebiotics is facilitating the development and/or metabolic activity of selected microbiota in the gut [16,17]. In the present study, we aimed to check whether prebiotics can ameliorate the status of fat-soluble vitamins and minerals in patients with CD on a GFD.

The most important finding of our study was an increase in plasma 25(OH)D concentration after 3 months of supplementation of a GFD with oligofructose-enriched inulin (Synergy 1). Vitamin D deficiency causes impaired mineralization of bone through parathormone action, which results in rickets in children and osteomalacia in adults [18,19]. An inverse correlation between vitamin D levels and the development of autoimmune, neurodegenerative, or cardiovascular diseases has also been reported [1,20]. The worldwide prevalence of vitamin D deficiency is estimated at 15% of the population [21], while this prevalence is higher in CD patients at diagnosis, ranging from 40 to 97% depending on the study [22,23]. Since vitamin D levels have been found to be negatively correlated with age [18], vitamin D deficiency is even more common in adult CD patients. A gluten-free diet improves vitamin D status only in a subset of CD patients. In recently published data, vitamin D deficiency was reported in 52% of children with newly diagnosed CD [22]. In a study by Mager et al. [24], a suboptimal level of 25(OH)D was noted in 43% of children and adolescents with CD at diagnosis, and a GFD diet decreased this prevalence by half. In our study, 88% of CD children had vitamin D deficiency or insufficient vitamin D levels at baseline. Supplementation with prebiotics resulted in an improvement of vitamin D status and, consequently, the number of children with a suboptimal level of vitamin D was reduced by half. It is well documented that prebiotics improve calcium metabolism in patients [7,8]. However, to our best knowledge, their effects on vitamin D status have only been assessed in animal studies. The use of xylooligosaccharides in poultry diet increased plasma concentration of 1,25-dihydroxyvitamin D3 [25]. The authors explained this phenomenon by an activation of 25-hydroxylase and 1-alpha-hydroxylase—enzymes involved in vitamin D metabolism.

Understanding the mechanisms responsible for an increase in vitamin D levels upon prebiotics supplementation is beyond the scope of this study. One possible explanation could be improved intestinal vitamin D absorption [16]. Prebiotics can stimulate short-chain fatty acid production by intestinal microbiota, which in turn facilitate gut epithelium functions [16]. An increase in short-chain fatty acid concentration after prebiotic intake in children with CD was confirmed in our previous study [17]. Of note, in this study, children of both experimental groups were consecutively supplemented with the stable dose of vitamin D before and during the intervention, but a significant increase in vitamin D level was observed only in the Synergy 1 group. Thus, the observed changes were not caused by differences in vitamin D intake.

Unlike the previous studies, which have shown a positive effect of prebiotics on calcium levels in non-CD individuals [7,8,13], and in spite of the well-known role of vitamin D in regulating calcium metabolism [26], our study did not demonstrate a significant effect of Synergy 1 on calcium serum status. This could be due to a small sample size. Interestingly, the number of children with hypocalcaemia at baseline (all of whom were asymptomatic) decreased from five to one child after Synergy 1 intake. However, these results are difficult to interpret in the absence of a sufficient number of children with baseline hypocalcaemia in the placebo group, and further studies are needed to assess the potential effect of prebiotics on calcium levels in children with CD.

Fat-soluble vitamins A and E are accumulated in the body; therefore, their deficiency is not commonly observed in healthy individuals [22]. It can be different in the case of diseases, like CD. Vitamin A deficiency is not commonly observed in CD and was estimated at 7–8% [22,23]. In our study, vitamin A deficiency (defined as vitamin A plasma concentration below 0.45 µmol/L; reference Mayo Clinical Laboratories) was not observed, and the concentration of this vitamin was not influenced by prebiotic administration. Vitamin E deficiency is more commonly observed, affecting 13.5% of CD children [22]. Vitamin E comprises tocopherols and tocotrienols with α-tocopherol being the most important antioxidant [27]. Vitamin E deficiency may occur in fat malabsorption, as the main natural sources of vitamin E are plant oils and nuts. It can result in severe neurological complications and in diseases with oxidative stress pathogenesis [27]. In our study, the supplementation of GFD with prebiotics had beneficial effects on vitamin E status, even though vitamin E deficiency (defined as a level <7.2 umol/L; reference Mayo Clinical Laboratories) was not observed in any of the study subjects. The effects of prebiotics on vitamin E have not been investigated to date, and further research is needed to explain these findings.

Importantly, our study was a double-blinded, placebo-controlled intervention and was conducted over a relatively long period of time, while most previous studies on the effect of prebiotics intake on calcium absorption followed participants over a period of a few days up to six weeks [8]. A few limitations of our study need to be mentioned. The sample size was not calculated due to the absence of primary outcome data and to the preliminary character of the study. However, the obtained results can be used to calculate the sample size for future fully powered studies. Another limitation is the small number of participants and wide range of age, which is also typical for a pilot and single-center study. Finally, no control group of healthy children was used in the study. However, the main purpose of this study was to compare the level of selected parameters before and after the administration of prebiotics in the same group of patients. Despite these limitations, this study is the first attempt to evaluate the effect of oligofructose-enriched inulin on fat-soluble vitamins status in CD children.

## 5. Conclusions

The presented findings show that oligofructose-enriched inulin (Synergy 1) added to the GFD essentially improves vitamin D and E status in children with CD. We regard that a monitoring of 25(OH)D level in children with CD on a GFD could be beneficial. The administration of Synergy 1 as a supplement to the GFD could be considered as a novel complementary method of management of fat-soluble vitamin deficiency in pediatric CD patients. However, further prospective multicenter cohort studies are needed to determine the dose–response and to optimize the effectiveness of supplementation of the GFD with Synergy 1.

## Figures and Tables

**Table 1:** Comparison of anthropometric parameters between Synergy 1 and placebo groups at the baseline.

	Synergy 1	Placebo	*p*-Value
Gender	F, 11; M, 7	F, 10; M, 6	0.797
Age [years]	9.8 ± 4.1	9.8 ± 4.3	0.945
Weight [kg]	36.6 ± 18.3	33.7 ± 16.2	0.809
Height [cm]	140.6 ± 23.6	136.8 ± 21.8	0.622
BMI [kg/cm^2^]	17.2 ± 3.5	17.0 ± 3.6	0.809

F—females; M—males; BMI—Body Mass Index

**Table 2:** Serum and urine measurements of minerals and intact parathormone (iPTH) at baseline (T0) and after intervention (T1). Quantitative variables with a normal distribution are expressed as mean ± SD; quantitative variables which showed a non-normal distribution are expressed as median (P25–P75).

	T0			T1				
	Placebo Group	Synergy 1 Group	*p*-Value	Placebo Group	Synergy 1 Group	*p*-Value	*p*-Value Placebo T0 vs. T1	*p*-Value Synergy 1 T0 vs. T1
	Serum							
Ca [mg/dL]	9.4 ± 0.3	9.2 ± 0.3	0.179	9.2 ± 0.3	9.3 ± 0.2	0.552	0.450	0.249
P [mg/dL]	4.7 (4.3–5.2)	4.5 (4.2–4.9)	0.469	4.8 (4.6–5.0)	4.6 (4.3–5.0)	0.250	0.169	0.932
Mg [mg/dL]	2.0 (1.9–2.2)	2.1 (2.0–2.2)	0.479	1.9 (1.9–2.1)	2.1 (2.0–2.1)	0.336	0.959	0.727
Total protein [g/dL]	7.5 (7.1–7.6)	7.4 (7.1–7.6)	0.843	7.1 (6.9–7.3) ^a^	7.2 ± 0.4	0.174	0.008	0.103
Albumin [g/dL]	4.2 (4.0–4.3)	4.2 (4.0–4.3)	0.809	4.1 (4.0–4.1) ^a^	4.2 (4.0–4.3)	0.357	0.041	0.307
	Plasma							
iPTH [pg/mL]	20.9 (12.0–33.4)	22.5 (15.6–31.1)	0.931	22.7 (12.7–30.1)	23.9 (14.4–29.0)	0.688	0.875	0.528
	Urine							
Ca [mg/mg Cr *]	0.07 (0.05–0.27)	0.09 (0.03–0.08)	0.605	0.08 (0.05–0.11)	0.09 (0.03–0.08)	0.890	0.593	0.925
P [mg/mg Cr]	0.85 ± 0.34	0.90 ± 0.41	0.448	1.06 ± 0.37 ^a^	0.90 ± 0.41	0.297	0.046	0.058
Mg [mg/mg Cr]	0.15 ± 0.06	0.15 ± 0.05	0.443	0.15 ± 0.07	0.15 ± 0.05	0.948	0.575	0.634

* Cr—creatinine. ^a^ —statistically significant differences within groups before and after intervention (*p* < 0.05).
